# Retro-priming, priming, and double testing: psi and replication in a test–retest design

**DOI:** 10.3389/fnhum.2014.00154

**Published:** 2014-03-18

**Authors:** Thomas Rabeyron

**Affiliations:** LPPL, Psychology Department, University of NantesNantes, France

**Keywords:** retro-priming, priming, anomalous experiences, precognition, PSI, replicability, methodology

## Abstract

Numerous experiments have been conducted in recent years on anomalous retroactive influences on cognition and affect (Bem, 2010), yet more data are needed to understand these processes precisely. For this purpose, we carried out an initial retro-priming study in which the response times of 162 participants were measured (Rabeyron and Watt, 2010). In the current paper, we present the results of a second study in which we selected those participants who demonstrated the strongest retro-priming effect during the first study, in order to see if we could replicate this effect and therefore select high scoring participants. An additional objective was to try to find correlations between psychological characteristics (anomalous experiences, mental health, mental boundaries, trauma, negative life events) and retro-priming results for the high scoring participants. The retro-priming effect was also compared with performance on a classical priming task. Twenty-eight participants returned to the laboratory for this new study. The results, for the whole group, on the retro-priming task, were negative and non-significant (*es* = −0.25, ns) and the results were significant on the priming task (*es* = 0.63, *p* < 0.1). We obtained overall negative effects on retro-priming results for all the sub-groups (students, male, female). Ten participants were found to have positive results on the two retro-priming studies, but no specific psychological variables were found for these participants compared to the others. Several hypotheses are considered in explaining these results, and the author provide some final thoughts concerning psi and replicability.

## Introduction

Non-ordinary mental expressions are frequently associated with altered states of consciousness (Cardeña, 2014) and potential specific interactions between mind and reality that are currently not explained by known physical or biological mechanisms, called *psi*. Although numerous studies have been conducted in order to prove the existence of these interactions (Radin, 2006; Tressoldi, 2011) or explain them using a more classical approach (Holt et al., 2012), the results of this research and its interpretations are still a topic of debate in the scientific community (Alcock et al., 2003).

Psi has been extensively studied during the last twenty years, most notably through the use of ganzfeld research (Bem and Honorton, 1994). In a typical ganzfeld study, a “sender” (situated in a shielded room) tries to influence a “receiver” (situated in the “ganzfeld” that is supposed to improve psi perceptions) in order to help him to visualize a target—usually a short movie. The receiver then has to choose between several movies (the target and three decoys), indicating which one was “sent.” This protocol has been replicated dozens of times and has produced significant and controversial results concerning the reliability of the effect observed (Milton and Wiseman, 1999; Bem et al., 2001; Storm and Ertel, 2001, 2002; Wackermann et al., 2008; Storm et al., 2010; Williams, 2011).

The details of this controversy will not be examined here; rather, we will note that one of the main difficulties in ganzfeld research, and more generally with what are called “free choice settings” (Storm et al., 2010), is that participants have to freely describe what they are thinking and feeling during the session. The participants and experimenters generally have difficulty in discriminating between the participant's imagination and supposedly “real” psi information. The latter could indeed be unconscious, and the description of the target would then be a mix of potential psi information perceived unconsciously and associations coming from several unconscious levels of mental functioning. This could explain the difficulty in obtaining stronger effect sizes in ganzfeld experiments.

This kind of observation has led to the development of the “presentiment paradigm,” in which experimenters test unconscious responses (Radin, 1997). Such an effect could be more reliable than usual conscious responses. In a basic presentiment experiment, participants' reactions are measured *before* they see neutral, violent, or erotic pictures (Radin, 2004). Researchers have thus, obtained small but significant differences in the intensity of reactions before the stimulus. The same kind of protocol has been carried out in different settings, for example, using sounds instead of pictures (May et al., 2005) or using image-priming, with smiling and angry faces (De Boer and Bierman, 2006). A recent meta-analysis produced significant results from presentiment experiments (Mossbridge et al., 2012).

Bem (2010) has more recently developed several paradigms concerning anomalous retroactive influence on cognition and affect, in an attempt to replicate this effect more globally and facilitate the replication process amongst several laboratories. One of these paradigms is a backward priming set-up called “retro-priming.” In a classical priming experiment, the participant's reaction is measured after he or she has seen the prime. In a retro-priming experiment, as in presentiment research, the participant's response time is measured not after but *before* the prime. The participant has to push a button to indicate if a picture is positive or negative. Then, the participant sees a prime that is a positive or a negative word. Response time is measured to find out if participants were influenced by the prime they saw after the picture.

In order to try to replicate this effect, we first carried out a retro-priming experiment in which we looked for correlations between anomalous experiences, psychological variables (mental health, mental boundaries, trauma, negative life events) and retro-priming results (Rabeyron and Watt, 2010). These results (see Table 1) were non-significant on the whole population (*n* = 162), but we obtained a slightly positive significant effect on the student population (*n* = 112; *r* = 0.17; *p* < 0.05), close to the effect size reported by Bem (*d* = 0.20) (2010). More surprisingly, we also obtained a strong effect with male participants (*n* = 45; *r* = 0.41; *p* < 0.01).

**Table 1:** **Experiment 1 retro-priming and priming results**.

		**Retro-priming**		**Priming**	
**Group**	***N***	***t***	***es***	***t***	***es***
Whole group	155	1.32	0.11	8.06	0.65^***^
Student group	112	1.77	0.17^*^	7.44	0.70^***^
Male group	45	2.73	0.41^**^	2.73	0.41^**^
Female group	110	0.07	0.01	6.69	0.64^***^

* P < 0.05;

** P < 0.01;

*** *P < 0.001*.

Bem's results gave rise to debates concerning methodological and experimental aspects in the field of psychology that go beyond the existence of psi (Lebel and Peters, 2011; Miller, 2011; Rouder and Morey, 2011; Wagenmakers et al., 2011; Pashler and Harris, 2012). Incidentally, psi research has historically been the source of such methodological and statistical questions (Rhine et al., 1966). Bem's paper spawned numerous attempts to replicate it (see e.g., Galak et al., 2012; Bem et al., submitted) and reflections on the difficulty of direct replications in psychology (Ritchie et al., 2012). This aspect has been associated more generally with debates concerning the “decline effect” in science (Schooler, 2011) and a potential “replication crisis” (Stroebe and Strack, 2014) especially in the fields of psychology and medical sciences (De Winter and Happee, 2013). Several researchers have proposed that large numbers of research findings could be false (Ioannidis, 2005), for a number of reasons, such as insufficient statistical power or questionable research practices (Simmons et al., 2011; Bakker et al., 2012; Francis, 2012).

Replicability in psi research is also a well-argued topic, and has led some researchers to argue that psi is actually different from the already familiar classical physical effects. One proponent of this kind of theory is Walter von Lucadou, with his Model of Pragmatic Information (MPI) (Lucadou, 1995), which is associated more generally with General or Weak Quantum Theory (Atmanspacher et al., 2002). These theories suppose that exact replication of a psi effect would eliminate or change this effect because psi would correspond to a “non-local correlation” that could not be used to predict results; this is called the “non-transmission” axiom (Lucadou and Romer, 2007). More recently, Dick Bierman (2008) proposed the Consciousness Induced Restoration of Time-Symmetry (CIRTS), in which problems of reproducibility are seen as the consequence of time paradoxes. These kinds of theories have fundamental implications and are at the core of numerous current debates in the field.

Last but not least, another difficulty encountered in psi research is the purported need to use high scoring participants for example, better results have been obtained with selected participants in ganzfeld studies (Storm et al., 2010); Some researchers think that only a small proportion of the population could produce consistently high scoring results (McMoneagle, 2000) and that it is possible to select participants using a test and re-test set up (Ertel, 2005, 2013). From this point of view, the difficulty in obtaining a reliable effect comes from the need to pre-select the participants, which is rarely done. Is it possible to select the high scoring psi subjects with a retro-priming experimental set-up? And can we find a psychological profile corresponding to these subjects?

In the present study, we tried to deal with several of these aspects. We decided to select the high scoring participants from the first retro-priming experiment (that is, those who had shown a strong retro-priming effect) and ask them to perform the retro-priming experiment again. If the high scorers in the first study did so by chance alone, then in the second study their performance would tend to regress to the mean (Mee and Chua, 1991), but if a genuine psi effect, with a sufficient effect size, was the cause of their high scoring in the first study, then they would tend to continue to score well on the re-test. We also tried to replicate the *post-hoc* findings from the first study, that is, the significant results with students and male participants, and find common psychological characteristics and a specific profile amongst the high scoring participants from both experiments.

## Methods

### Participants

For the first experiment, 162 participants were recruited: 31 from a general population volunteer panel in Edinburgh University Psychology Department, 114 students from Edinburgh University's intranet website and 17 other participants from advertisements in shops and several internet websites. There were more females (71.6%) than males in the whole group. The median age was 28.64 years (range = 16−76). After the analysis of the first experiment's results, the participants with the most positive results on the psi task (which meant that their retro-priming results—the total logarithm response time of incongruent minus congruent trials—were more than 0.05; this applied to 39 participants, 23% of the whole group) were invited to a second study. Twenty-eight participants responded positively to our request and came back to the laboratory. There were more females (64.29%) than males (10 males and 18 females) and the median age was 26.07 years (range = 18−76).

### Retro-priming experiment

The psi task was a retroactive priming task devised by Bem (2008), as described further below. The computer used was a Dell Optiplex 745, running Windows XP. The program used for the psi task was designed by Daryl Bem at Cornell University with REAL basic. It was a slightly different version of the software than the one used by Bem (2008); this version used pictures as primes instead of words. We used a Windows version of this software, using an algorithm to generate a random sequence of numbers.

This psi task was a precognitive experiment in which the response time of participants was measured in order to see if they would be influenced by a prime (a picture) that they would see not before but *after* a word. Participants were shown a word on each of 64 trials and were asked to press one of two keys on the keyboard as quickly as they could, in order to indicate whether the word was pleasant or unpleasant. The participant's response time in making this judgment was the major dependent variable, and the difference in mean response times between incongruent and congruent trials was the index of a priming effect, with positive differences denoting faster responses to congruent trials. The first 32 trials constituted the retroactive priming procedure, and participants were told that a picture would be flashed on the screen just after they made their decision. In this condition, when the participant has a positive result, it appears as though he or she has been “influenced” by the picture seen after the word. A participant who is very permeable to psi information is expected therefore to obtain a very positive score. The remaining 32 trials constituted the standard “forward” priming procedure, and participants were told that from this point on, the flashed picture would appear before rather than after they made their response. The standard priming condition was used to allow us to compare psi results with a classical priming effect, and also to see if we would find correlations between priming results and other variables.

Response times shorter than 250 ms or longer than 2500 ms were regarded as outliers and were excluded from the data analysis, as were trials on which the participant made an error in judging the picture to be pleasant or unpleasant. Finally, because response-time data were positively skewed, all response times were log-transformed prior to being combined and analyzed. Shown below is the time sequence of events for Forward Priming and Retroactive Priming trials, respectively.

**Table:** 

**FORWARD PRIMING TRIAL**						
**Stimulus**	**Fixation spot**	**Picture (prime)**	**Blank**	**Word**	**Starry sky**	
Time (ms)	1000	150	150	Response time	2000	
**RETROACTIVE PRIMING TRIAL**						
**Stimulus**	**Fixation spot**	**Word**	**Blank**	**Picture (prime)**	**Blank**	**Starry sky**
Time (ms)	1000	Response time	300	500^1^	1000	2000

### Procedure

Two months after the first experiment, the participants who had been selected received an email asking them to participate in the experiment for a second time at the Psychology building. When they came back to the laboratory, it was explained by the principal investigator (the same than in the first study: Thomas Rabeyron, male, 29, open to the existence of psi), that they had been selected for this second study because of their high score in the first study. They were then invited to participate again in the task they had already completed during the first study. Finally, they were briefly interviewed about items they had marked as being true on the *Anomalous Experience Inventory* during the first study (Gallagher et al., 1994). They were also asked if anomalous experiences were important in their life. This short interview lasted an average of 10 min. Participants were given £5 in appreciation of their time and effort and they were told they would receive global and personal results by email when all the data had been analyzed. The study was approved by the Department of Psychology's ethics panel.

## Results

### First experiment

The results of the first experiment are available in Table 1 and, as already mentioned, can be found in a more detailed analysis in a previous paper (Rabeyron and Watt, 2010). During this first study, the results on the retro-priming task were non-significant (*t* = 1.32, *df* = 154, *p* = 0.09, *es* = 0.11) while the results on the priming task were significant (*t* = 8.06, *df* = 154. *p* < 0.001, *es* = 0.65). We then decided to create several groups during a *post-hoc* analysis, using sex and population as variables. We found that the student group had significant results on retro-priming and we found a negative correlation on the whole group between age and psi results, which could have explained the non-significant retro-priming results in the whole population. We also found an unpredicted and strong effect size in the male group (*n* = 45; *r* = 0.41; *p* < 0.01).

### Second experiment

The 28 participants who agreed to come back for the second experiment had obtained very significant results on the retro-priming task (*t* = 10.99, *df* = 27, *es* = 2.08) during the first study. Their results for the second experiment are available in Table 2.

**Table 2:** **Experiment 2 retro-priming and priming results**.

**Group**		**Retro-priming**		**Priming**	
	***N***	***t***	***r***	***t***	***r***
Whole group	28	−1.35	−0.25	3.35	0.63^**^
Student group	21	−1.39	−0.30	3.08	0.67^*^
Male group	10	−0.56	−0.18	4.30	1.36^**^
Female group	18	−1.23	−0.29	1.73	0.41

* P < 0.05;

** *P < 0.01*.

During this second study, analyses on the whole group (*n* = 28) demonstrated negative and non-significant results on the retro-priming task (*t* = −1.35, *df* = 27, *ns*, *es* = −0.25) and the results were significant on the priming task (*t* = 3.35, *df* = 27, *p* < 0.01, *es* = 0.63). For the student group (*n* = 21), the results were also negative and non-significant on the retro-priming task (*t* = −1.39, *df* = 20, *ns*, *es* = −0.30), but significant on the priming task (*t* = 3.08, *df* = 20, *p* < 0.05, *es* = 0.67). Again, for the male group (*n* = 10), the results were negative and non-significant for the retro-priming task (*t* = −0.56, *df* = 9, *ns*, *es* = −0.18) but significant for the priming task (*t* = 4.30, *df* = 9, *p* < 0.01, *es* = 1.36). Finally, for the female group (*n* = 18), there was a negative and non-significant effect on the retro-priming task (*t* = −1.23, *df* = 17, *ns*, *es* = −0.30) and there was no significant effect on the priming task (*t* = 1.73, *df* = 17, *ns*, *es* = 0.41). Overall, we found a significant negative correlation between the retro-priming results of the first and second studies (*r* = −0.46, *p* < 0.05) and a positive but non-significant correlation between the priming results of the first and second studies (*r* = 0.19, *ns*). The correlation between the priming and retro-priming results was non-significant (*r* = −0.045, *ns*).

### Psychological profiles of the best participants

Ten participants were found to have positive results (that is, showing a retro-priming effect) on the two retro-priming studies. Group comparisons between these 10 participants (six male and four female; mean age = 27.6 years) and the other 152 participants did not demonstrate significant differences on the psychological characteristics already used in the first study; that is, paranormal experiences (*U* = 609, ns, two-tailed), mental boundaries (*U* = 651.5, *ns*, two-tailed), mental health (*U* = 679.5, *ns*, two-tailed), childhood trauma (*U* = 492.5, *ns*, two-tailed) or negative life events (*U* = 521.5, *ns*, two-tailed).

From the interviews conducted with participants after the second study, it appeared that the highest scoring participant was a young female student in law who was very interested in the paranormal and who mentioned she was aware of events before they happen on some occasions. Among the 10 highest scoring participants, 5 (50%) said they have had precognitive experiences in the past. They had a range of beliefs concerning the paranormal, with some of them describing themselves as “believers” and others as “skeptics.”

## Discussion

### Retro-priming and replicability

While we had nearly significant results during the first study (Rabeyron and Watt, 2010), and more precisely a significant retro-priming effect size for the student group (*r* = 0.17), close to the one reported by Bem (*d* = 0.25) (2010), the results of this second study were non-significant. We also obtained overall negative effects on retro-priming results for all the groups (male, female, student, whole) and did not manage to replicate the strong effect size obtained (*r* = 0.41) with male participants in the first study. In previous research concerning psi (Ertel, 2005) some authors have suggested that psi effects could be reliable enough to maintain significant results in test–retest settings but we didn't manage to obtain such results in our retro-priming studies.

In terms of explaining this, we first need to take into account the potential impact of the regression to the mean effect (Mee and Chua, 1991; Kahneman, 2011)^2^. When best participants are selected during a first measurement, they will tend, by chance alone, to be close to the average during a second measurement. The more the results of the first measurement are the consequence of chance alone, the more the regression to the mean effect will be important. This effect would naturally lead to a decrease of the effect size in the second study, which is the case in our data. We used the technique proposed in Barnett et al. (2005) in order to evaluate the regression to the mean effect (rtme) on the results of the first study, and obtained a rtme = 0.15. The retro-priming results of the second study were still non-significant even when we took into account this regression to the mean effect (*r* = −0.10). These non-significant results would rather support a skeptical interpretation of psi data (Alcock, 2003; Wiseman, 2010; Wagenmakers et al., 2011).

These non-significant results could also be the consequence of a setting that was slightly different from that used in Bem's studies: we used pictures, rather than words, as primes, and we cannot know what impact (if any) this change had on the results, or if there was a habituation effect between the two studies. Additionally, we do not know if the fact that participants were told they had positive results on the first psi study had a potential negative impact on their results.

Finally, concerning the selection of high scoring participants, we did not find a typical psychological profile (for anomalous experiences, mental boundaries, mental health and childhood trauma, and negative life events) of the high scoring participants (that is, the 10 participants who had significant results for both studies). We note that half of the selected subjects described having had precognitive experiences in the past, but this qualitative analysis would need to be confirmed in future studies and is difficult to evaluate without a group control comparison.

### Priming, psi research, and replicability

The priming results from the first and the second studies showed a small and non-significant correlation (*r* = 0.19, *ns*), which echoes a recent paper by Cesario (2014) concerning the difficulty encountered in the replication of the priming effect. He explains:

“When researchers do not get the “right” combinations of variables, the failures end up in the file drawer. Indeed, this might be what is meant when researchers talk about having “insight” or “intuition” in conducting priming experiments in which they cannot verbalize why they made a decision but knew to make it” (p. 44).

It seems that the replicability difficulties we encountered here concern not only our retro-priming results, but also our priming results. They also contribute more globally to current debates, mentioned in the introduction, on replicability and the decline effect (Schooler, 2011; Francis, 2012). We might ask, then, what are the origins of these difficulties, and are they the same as the ones encountered more globally in the field of psychology? Are they the consequence of variations in the way replications are conducted (Simmons et al., 2011) and do we need to use new statistics (as proposed notably by Cumming, 2014)?

Along similar lines, Cesario's thoughts on the “insight” or “intuition” involved in conducting research and specifically on replicability difficulty have also been a regular topic in psi research (Kennedy, 2003, 2004, 2013; Etzold, 2006; Hyman, 2010). Some psi researchers have thus, proposed the idea that the replication of a psi effect would actually, in some conditions, *suppress* it and may even cause *negative results*. Indeed, we obtained a negative and significant correlation between the results of the first and second studies (*r* = −0.46, *p* < 0.05). We cannot draw firm conclusions concerning this effect because it may partly be due to the regression to the mean effect, as already mentioned, and from an Occam's razor point of view it would of course be more pertinent to view it as evidence of the absence of psi. But this kind of decline effect is not only a topic of debate in psychology (Schooler, 2011) but is also extremely common in psi research: Bierman (1980) describes it as “negative reliability,” Beloff (1994) speaks about psi as being “actively evasive,” Pallikari and Boller (1997) mention a “balancing effect” between positive and negative replication and Hansen (2001) has proposed a broader theory called “the trickster” to explain these kind of negative results.

We could consider these explanations as kind of “auto-immune” responses from psi researchers when they obtain negative results. Nevertheless, on carefully examining the data, patterns are often noticed that are difficult to explain by chance alone (Lucadou and Romer, 2007), and some theorists have tried to take these strange variations into account in explaining their results. Thus, the MPI (Lucadou, 1995), associated more generally with General or Weak Quantum Theory (Atmanspacher et al., 2002; Filk and Römer, 2011), predicts such sign inversion if researchers attempt to replicate psi effects, and these kinds of results have already been obtained in numerous previous experiments (Lucadou and Romer, 2007). From the MPI perspective, psi effects are considered as “non-local correlations” that share several characteristics, from a metaphoric point of view, with entanglement correlations at a quantum level. These correlations would be produced in systems with an organizational closure (a concept introduced notably by Varela et al., 1974 concerning the way a system is organized) and a psi experiment could be an example of this kind of system. Several parameters (such as “documentation” and “motivation” for example) could increase or decrease the organizational closure of a system and consequently produce (or not) a psi effect.

From this perspective, in our first study (which was already a replication of Bem's studies), we would have obtained a “displacement effect” with a strong and unexpected effect on the male population. Then, in the second study, we tried to use the retro-priming effect to transmit and extract information (a prediction of this effect) from the system (the experiment) which would suppress the effect that we wanted to replicate (especially the strong effect size with male participants). Consequently, as predicted by the MPI, we would have obtained in the second study a sign inversion and a disappearance of the initial *post-hoc* finding, which means more precisely that we had enough motivation to produce psi but the sign had to change in order to allow the expression of a psi effect without breaking the “non-transmission axiom” (NT-Axiom) (Lucadou and Romer, 2007). Of course, this interpretation is only a *post-hoc* analysis, but (Lucadou and Romer, 2007) proposes several ways of testing these kind of processes. More recently, Bierman (2008) also proposed a general model, the CIRTS, in which decline effects are explained as a consequence of the time paradox. In CIRTS, psi effects are seen as a fundamental ability of consciousness to partially restore time-symmetry. The retro-priming effect could be a consequence of such a principle, which more generally could have implications for the global and coherent synchrony of brain processes. Bierman predicted more precisely that any attempt to increase the effect size in a presentiment experiment, as in our retro-priming research, would fail because of these subtle time paradoxes that could be studied and tested with several experimental set-ups (Bierman, 2008).

The data we have obtained here does not allow us to draw conclusions concerning the relevance of these different models, but these results echo previous patterns frequently reported in research aiming for the replication of psi processes. New insights with regards to psi and retro-priming should take these hypotheses into account. If they were to be true, they could indeed have important implications for experimental and methodological psychological design. Future research should focus on improving the reliability and replicability of retro-priming studies (with, for example, the use of prospective meta-analysis, see, e.g., Kennedy, 2013) and priming studies (Cesario, 2014). It should also aim to develop theoretical models that allow empirical predictions, as proposed by Lucadou and Romer (2007) and Bierman (2008), in order to demonstrate if these patterns are mere cognitive illusions or if they are a real aspect of supposed psi effects.

### Conflict of interest statement

The author declares that the research was conducted in the absence of any commercial or financial relationships that could be construed as a potential conflict of interest.

## Footnotes

^1^The prime is displayed longer in the retro-priming trials because the prime appears after a blank screen and it might be missed if it appeared too briefly. The aim is also to enhance in this way the potential retro-priming effect.

^2^Comparatively, classical priming results have not been influenced by the regression to the mean effect: the high scoring participants were selected only on their retro-priming results for the second study and not on their priming results. This could explain why the results of priming results were quite similar between the first (*r* = 0.65) and the second study (*r* = 0.63).
